# Assessing the Use of Neonatal Sepsis Guidelines and Antibiotic Prescription With Large-Scale Prospective Data From Zimbabwe and Malawi

**DOI:** 10.1093/jpids/piaf017

**Published:** 2025-02-21

**Authors:** Nushrat Khan, Gwendoline Chimhini, Som Kumar Shrestha, Mario Cortina-Borja, Simbarashe Chimhuya, Gloria Zailani, Hannah Gannon, Marcia Mangiza, Felicity Fitzgerald, Michelle Heys, Msandeni Chiume

**Affiliations:** Department of Primary Care and Public Health, Imperial College London, London, United Kingdom; Department of Child and Adolescent and Women’s Health, Faculty of Medicine and Health Sciences, University of Zimbabwe, Harare, Zimbabwe; Sally Mugabe Central Hospital Neonatal Unit, Harare, Zimbabwe; Department of Population Health, London School of Hygiene and Tropical Medicine, London, United Kingdom; Human Development Report Office, United Nations Development Programme, New York, NY, United States; Population, Policy and Practice Research and Teaching Department, University College London Great Ormond Street Institute of Child Health, London, United Kingdom; Department of Child and Adolescent and Women’s Health, Faculty of Medicine and Health Sciences, University of Zimbabwe, Harare, Zimbabwe; Sally Mugabe Central Hospital Neonatal Unit, Harare, Zimbabwe; Department of Paediatrics, Kamuzu Central Hospital, Lilongwe, Malawi; Population, Policy and Practice Research and Teaching Department, University College London Great Ormond Street Institute of Child Health, London, United Kingdom; Sally Mugabe Central Hospital Neonatal Unit, Harare, Zimbabwe; Department of Infectious Disease, Imperial College London, School of Medicine, South Kensington Campus, London, United Kingdom; Population, Policy and Practice Research and Teaching Department, University College London Great Ormond Street Institute of Child Health, London, United Kingdom; Department of Paediatrics, Kamuzu Central Hospital, Lilongwe, Malawi

**Keywords:** Low resource setting, diagnostics, neonatal sepsis, antimicrobial stewardship

## Abstract

**Background:**

Neonatal sepsis is a major cause of mortality in low-resource settings. We assessed how neonatal sepsis guidelines were used in 2 Zimbabwean hospitals and 1 Malawian hospital.

**Methods:**

Using routine data collected with the digital health intervention, Neotree, we retrospectively reviewed doctors’ and nurses’ agreement with national and World Health Organization (WHO) guideline recommendations for antibiotic prescription for sepsis. We compared clinical features and outcomes of neonates who should have received antibiotics as per guideline with those who actually received them and fitted a logistic regression model to identify features associated with prescription.

**Results:**

Data were collected between January 2021 and June 2022 from 10 868 neonates: 6045 admitted to Sally Mugabe Central Hospital (SMCH), 1094 to Chinhoyi Provincial Hospital (CPH) and 3729 to Kamuzu Central Hospital (KCH). Complete implementation of national guidelines would increase antibiotics at admission: from 2188 (38%) to 3745 (64%) at SMCH, 472 (44%) to 852 (79%) at CPH, and 1519 (41%) to 3043 (82%) at KCH. Clinical features of sepsis were frequently not acted on, but the case fatality rate was lower in those not prescribed antibiotics despite guideline recommendation. Application of WHO guidelines would increase antibiotic prescription to 91% at SMCH, 88% at CPH, and 77% in KCH. Maternal risk factors for sepsis, male gender, low birth weight, older age at admission, and spontaneous vaginal delivery were associated with higher rate of antibiotic prescription.

**Conclusions:**

Guideline-recommended clinical signs for sepsis are inconsistently used, with clinicians using other features for antibiotic decision-making. Work is needed to revise clinical diagnostic algorithms in low-resource settings to ensure they are useful, usable and contextually appropriate.

## Teaser

Using routinely collected healthcare data from >10 000 newborns at 3 sites in sub-Saharan Africa, we demonstrate that were neonatal sepsis national/international guidelines to be consistently followed, antibiotic prescription rates would increase by 40%-50%. Clinicians appear to use other clinical features to guide prescription.

## INTRODUCTION

Worldwide, neonatal sepsis causes ~800 000 deaths annually, the majority of which are in low-resource settings (LRS).^1,2^ Gold standard diagnosis of sepsis uses blood cultures, which take at least 24-48 h to process and depend on laboratory facilities and staff. The initial decision of clinicians to manage neonates with suspected sepsis is thus taken on the basis of clinical signs and risk factors. This decision has critical implications: a missed diagnosis of sepsis can result in death, so clinical guidelines in LRS prioritize sensitivity over specificity.^3,4^ This means that many neonates are referred, admitted, and treated with antibiotics unnecessarily, generating antimicrobial resistance, which is rapidly expanding in neonatal units in LRS.^5,6^ These neonates take up space and resources in LRS healthcare facilities, often causing considerable out-of-pocket expenses for their families. Overuse of antibiotics also compounds local drug stockouts, impacting treatment for neonates at the highest risk.^7^ Furthermore, the antimicrobials themselves are not without risk: there is evidence that antibiotic use in preterm neonates impacts the development of the gut microbiome, predisposing to necrotizing enterocolitis in the short term and conditions such as asthma in the longer term.^8^

The World Health Organization (WHO) guidelines use one of 7 clinical signs to identify possible serious bacterial infection (PSBI, incorporating pneumonia, meningitis, and sepsis) in symptomatic neonates,^9^ derived from a study carried out from 2004 to 2005.^10^ Recent studies question their reliability: prospective evaluation found ten-fold variations in incidence between countries with similar demographics^11^ which raises the possibility that variations may be due to differing interpretations of clinical signs. Signs are given equal weight despite not carrying equal risk: population-based prospective surveillance found that neonates with poor feeding had a >40-fold higher mortality risk than those with fever.^12^

Adding a level of complexity, many countries have national guidance on the identification of sepsis. In Zimbabwe, the Essential Drugs List in Zimbabwe 2020 (EDLIZ 2020)^13^ Neonatal sepsis guidelines are used, (Supplementary Table 1a) while in Malawi, the Care of the Infant Newborn (COIN 2017)^14^ guideline is used (Supplementary Table 1b), in addition to WHO guidelines (See Supplementary Appendix for details on antimicrobial use at study sites).

It is unclear what impact recent epidemiological and health systems changes, such as the predominance of resistant Gram-negative infections,^15^ and a huge shift from home-based to hospital-based deliveries^16^ respectively, will have had on the use and utility of WHO and national guidelines for identification of neonatal sepsis.

The Neotree system, including bedside application (app) has been implemented in Sally Mugabe Central Hospital (SMCH), Harare, Zimbabwe since November 2018, in Kamuzu Central Hospital (KCH), Lilongwe, Malawi^17,18^ since April 2019, and in Chinhoyi Provincial Hospital (CPH) in Chinhoyi, Zimbabwe since December 2020. The app contains clinical decision support and data capture functions, providing guidance and suggested diagnoses alongside replacing paper admission and discharge/death forms.

We aimed to assess the extent to which national and international guidelines for identifying sepsis were adhered to in practice and to compare neonates who received antibiotics as per guidelines with those who met the criteria but were not prescribed them.

## METHODS

### Study Population

Sally Mugabe Central Hospital is a public referral hospital in Harare, Zimbabwe with ~12 000 deliveries a year. The 100-cot neonatal unit accepts ~3000 admissions per year, including nationwide surgical referrals. Care is mainly doctor-led, with junior doctors admitting babies and senior overview at ward rounds. Chinhoyi Provincial Hospital has ~1000 deliveries annually with a 20-cot neonatal unit, care being led by nurses. Kamuzu Central Hospital is a nationwide referral hospital in Malawi, with a 75-cot unit where care is mainly nurse-led. At all sites, it will be the admitting nurse or junior doctor who makes the initial decision to commence antimicrobials. Hard copies of national guidelines and risk factors are available on all units (Supplementary Appendix). Since implementation, all neonates admitted at these units have been eligible for inclusion in Neotree: using the app for admission and discharge documentation is routine practice. As neonates with a guarded prognosis are managed supportively rather than actively at SMCH, these neonates were excluded from analysis. These neonates are identified by the admitting doctor to have a condition so severe as to be incompatible with more than a few hours of life. For this study, we used data collected between January 2021 and June 2022—from the timepoint of data collection being streamlined at CPH to the starting time of conducting this data analysis.

### Data Collection

Data collection, management, and storage within Neotree have been described elsewhere.^18,19^ Neotree guides assessment of the neonate and collects data on patient demographics, examination findings, diagnoses, and interventions. Data are captured prospectively at admission and at discharge/death. At the time of this study, only emergency resuscitation algorithms as per Helping Babies Breathe were activated within the app.

### Outcomes

1. Decision to prescribe antibiotics at admission, specifically first-line antibiotics for bloodstream infection: crystalline penicillin and gentamicin; ceftriaxone and metronidazole for neonates with surgical conditions, or other antibiotics prescribed in case of stockouts (eg, ampicillin replacing crystalline penicillin).
2. Mortality: captured as “neonatal death” in the discharge/death form. All other outcomes, including discharge, transfers out to other hospital and discharged on parental request, were considered alive.

### Data Analysis

For each country, we identified the variables within Neotree that corresponded to a clinical feature consistent with sepsis, as per guideline in each country. The WHO PSBI guideline and WHO risk factors for sepsis were applied to data from both countries. For some variables, there was not an exact match between the Neotree and the guideline, eg, we linked the WHO clinical feature of “reduced movement or no movement at all” with “lethargy” or “coma” in Neotree. In Zimbabwe, we included the weighting given to “major” and “minor” features and the guideline discrimination between severe respiratory distress at birth in a term infant vs “delayed transition to extra-uterine life”: mild or moderate respiratory distress improving with time (Supplementary Appendix). We quantified the number of neonates that would be eligible for antibiotic treatment for sepsis or PSBI as per guideline. This was compared with those for whom the admitting clinician prescribed antibiotics at the time of admission. We then estimated case fatality rates (CFR) in both groups.

Clinical signs, clinician-documented preliminary diagnosis at admission, risk factors, and CFRs were compared between groups receiving antibiotics and those not receiving them but eligible using Chi-squared tests for categorical variables.

Nine variables that are likely to be associated with antibiotic prescriptions were selected based on clinical knowledge (**Table 1**).^12,20^ In case variables had <1% missing values, those rows were excluded from the dataset for modeling purposes (*n = *34) based on assumption that exclusion of 0.003% cases has minimal effect on the model. Variables with >1% missing values were imputed. Numeric explanatory variables (age and birth weight) were assessed to ascertain whether data were missing at random or not at random and imputed using medians conditional on diagnoses at admission and gestational age. Generalized linear modeling was used to determine the effect that the selected a priori variables had on the probability that a neonate had received antibiotics. Collinearity between variables was tested using generalized variance-inflation factors.^21^ Due to high collinearity between gestation age and birth weight, the former was excluded from the final model as birth weight experiences less error measurement in these settings. Gender was also excluded as there was no association with antibiotics prescription. Logistic regression models were fitted for the final set of eight variables using a stepwise approach based on Akaike’s Information Criterion (AIC) as a goodness-of-fit statistic.

**Table 1.** Demographics of 10 868 Neonates Admitted between January 2021 and June 2022 to SMCH in Harare and Chinhoyi, Zimbabwe and KCH, Lilongwe, Malawi, Including Prescription of Antibiotics at Admission. *P-*values <0.05 highlighted in bold.

Predictor	Levels	Total (*n = *10 868)	Received antibiotics (*n = *4273)	Did not receive antibiotics (*n = *6595)	Crude Odds Ratio (95% CI)	*P*-value
Age at admission (hours) median [Q1-Q3]	–	3.0 [1.0-17.00]	4.0 [1.00-33.0]	3.0 [1.0-11.0]	1.0018 (1.0015-1.0021)	**<.001**
	Missing *n* (%)	124 (1.14)	48 (38.7)	76 (61.3)	–	–
Sex	Male	5997 (55.2)	2428 (56.82)	3569 (54.12)	Reference	–
	Female	4851 (44.6)	1841 (43.08)	3010 (45.64)	0.897 (0.83-0.97)	**.007**
	Unsure	20 (0.2)	4 (0.09)	16 (0.24)	0.366 (0.105-0.999)	.072
Birthweight (grams) median [Q1-Q3]		2500 [1730-3090]	2500 [1700-3000]	2500 [1790-3100]	0.99989 (0.99985-0.9999)	**<.001**
	Missing *n* (%)	240 (2.2)	114 (47.5)	126 (52.5)	–	–
Estimated Gestational age (weeks) median [Q1-Q3]		37.00 [33.0-39.0]	37.00 [33.0-39.0]	37.00 [34.0-39.0]	0.975 (0.966-0.984)	**<.001**
	Missing *n* (%)	3 (0.03)	–	3 (100)	–	–
Mode of delivery	Vaginal delivery	7357 (67.7)	3142 (73.53)	4215 (63.91)	Reference	–
	Elective cesarean section	620 (5.7)	171 (4.0)	449 (6.81) (67.94)	0.511 (0.425-0.613)	**<.001**
	Emergency cesarean section	2462 (22.7)	817 (19.12)	1645 (24.94)	0.665 (0.604-0.732)	**<.001**
	Other (forceps, vacuum, breech, induced vaginal delivery)	422 (3.88)	142 (3.32)	280 (4.25)	0.673 (0.546-0.827)	**.0002**
	Missing *n* (%)	7 (0.06)	1 (0.02)	6 (0.09)	–	–
Place of birth	Inborn	7153 (65.8)	2492 (58.32)	4661 (70.67)	0.662 (0.603-0.728)	**<.001**
	Transferred in from another facility	735 (6.76)	374 (8.75)	361 (5.47)	1.28 (1.085-1.51)	**.0035**
	Born in the community	558 (5.13)	327 (7.65)	231 (3.50)	1.755 (1.456-2.119)	**<.001**
	Born outside but birthplace unknown	2422 (22.3)	1080 (25.27)	1342 (20.35)	Reference	–
Maternal Age (years) median [Q1-Q3]		25.0 [21.0-31.0]	25.0 [20.0-31.0]	25.0 [21.0-31.0]	0.991 (0.985-0.997)	**.003**
	Missing *n* (%)	31 (0.29)	4 (12.9)	27 (87.1)	–	–
Maternal HIV infection	No test taken/unknown	1608 (14.8)	662 (15.49)	946 (14.34)	Reference	–
	Reactive	871 (8.01)	372 (8.71)	499 (7.57)	1.064 (0.899-1.258)	.47
	Nonreactive	8389 (77.2)	3239 (75.8)	5150 (78.09)	0.893 (0.81-0.996)	**.04**
Maternal risk factors of sepsis	Prolonged rupture of membranes >18 hours	654 (6.02)	455 (10.65)	199 (3.02)	4.285 (3.613-5.098)	**<.001**
	Foul Liquor	277 (2.55)	189 (4.42)	88 (1.33)	4.025 (3.125-5.223)	**<.001**
	Fever >38 during labor	51 (0.47)	27 (0.63)	24 (0.36)	2.108 (1.214-3.683)	**.008**
	Prolonged 2^nd^ stage	180 (1.66)	92 (2.15)	88 (1.33)	1.959 (1.458-2.634)	**<.001**
	Born before arrival	475 (4.37)	298 (6.97)	177 (2.68)	3.155 (2.61-3.824)	**<.001**
	No risk factors	9231 (84.9)	3212 (75.17)	6019 (91.27)	Reference	–
Hospital site	Chinhoyi Provincial hospital (Zimbabwe)	1094 (10.1)	477 (43.6)	617 (56.4)	Reference	–
	Sally Mugabe Central hospital (Zimbabwe)	6045 (55.6)	2277 (37.67)	3768 (62.33)	0.79 (0.69-0.89)	**.0003**
	Kamuzu Central hospital (Malawi)	3729 (34.3)	1519 (40.73)	2210 (59.27)	0.89 (0.78-1.02)	.096
Neonatal outcome	Discharged	8027 (73.9)	2924 (68.43)	5103 (77.38)	Reference	–
	Died	1901 (17.5)	999 (23.38)	902 (13.68)	1.938 (1.752-2.144)	**<.001**
	Outcome missing	940 (8.65)	350 (8.19)	590 (8.95)	1.046 (0.908-1.202)	.531

All analyses were performed in the R statistical language (version 4.1.2) on R Studio.^22^

## RESULTS

In total, 10 868 neonates were included in the analyses, 6045 admitted to SMCH, 1094 to CPH, and 3729 to KCH. Ninety-nine babies with a guarded prognosis (as described above) were excluded from SMCH data. Age is needed to decide on the presence of EDLIZ major/minor criteria, therefore, 68 neonates from SMCH and 10 neonates from CPH with missing age were excluded for this part of the analysis, leaving 5878 and 1084 neonates respectively. Demographics are presented in **Table 1**. For modeling purposes, 3 records with missing gestation age and 31 records with missing maternal age were excluded, resulting in 10 834 records for the final model (**Table 5**).

**Table 5.** Association between Antibiotic Prescription and Predictor Variables, Including Regression Coefficients and Adjusted Odds Ratios for the Final Model (*n = *10 834). *P-*values <0.05 highlighted in bold.

Predictor	Estimate	Standard error	Odds ratio	95% CI	*P*-value
Intercept	−0.28	0.15	0.76	0.57-1.01	.061
Birthweight (natural spline 1)	0.09	0.21	1.09	0.72-1.65	.688
Birthweight (natural spline 2)	−0.47	0.16	0.62	0.45-0.86	**.004**
Age	0.002	0.0002	1.0017	1.001-1.002	**<.001**
Female	−0.1	0.042	0.9	0.83-0.88	**.014**
Gender unsure	−1.19	0.586	0.3	0.08-0.88	**.04**
Elective cesarean	−0.38	0.098	0.68	0.56-0.83	**.001**
Emergency cesarean	−0.23	0.054	0.79	0.72-0.88	**<.001**
Other mode of delivery	−0.36	0.11	0.7	0.56-0.87	**.002**
Inborn	−0.36	0.05	0.7	0.63-0.78	**<.001**
Born in the community	−0.098	0.14	0.91	0.69-1.19	.48
Transferred from other health facility	0.09	0.09	1.09	0.92-1.3	.31
Maternal fever	0.78	0.29	2.18	1.24-3.85	**.007**
Offensive liquor	1.65	0.13	5.27	4.03-6.89	**<.001**
Prolonged rupture of membrane	1.62	0.09	5.04	4.23-6.03	**<.001**
Prolonged 2nd stage	0.79	0.16	2.22	1.63-3.03	**<.001**
Born before arrival	0.98	0.14	2.65	2.0-3.52	**<.001**
Admitted to KCH	−0.34	0.09	0.71	06-0.84	**.0001**
Admitted to SMCH	−0.45	0.08	0.64	0.54-0.75	**<.001**
Missing outcome	−0.08	0.18	0.92	0.64-1.31	.65
Neonatal death	0.29	0.21	1.35	0.96-1.88	.08
Admitted to KCH: Missing outcome	−0.09	0.23	0.91	0.57-1.43	.67
Admitted to SMCH: Missing outcome	0.22	0.21	1.25	0.83-1.87	.29
Admitted to KCH: Died	0.09	0.19	1.09	0.75-1.59	.65
Admitted to SMCH: Died	0.68	0.19	1.97	1.37-2.83	**.0003**

Overall CFR per 1000 admissions was 185 (95% CI, 177-194). Site-specific CFR was 172 (95% CI, 161-183) at SMCH, 201 (95% CI, 173-232) at CPH, and 202 (95% CI, 187-217) at KCH. A total of 940 neonates with missing outcomes were excluded when calculating these CFRs as these babies could have been discharged or had other outcomes. Among those who had missing outcomes, the percentage of those who received antibiotics was similar to those who did not. This group was included as a missing outcome category in the model to reduce potential bias.

### Zimbabwe (SMCH)

At SMCH 38.7% (*n = *2277) received antibiotics, in comparison to 92.1% (*n = *5411) according to WHO guidelines and 63.8% (*n = *3745) eligible as per EDLIZ 2020, where a neonate with at least one major risk factor/clinical feature, meconium aspiration or 2 or more minor risk factors/clinical features present is eligible for antibiotics (**Table 2a**). Among those deemed eligible according to the local guidelines, 48.6% (*n = *1821) received antibiotics (major and minor criteria combined). An additional 17.2% (*n = *367) received antibiotics despite ineligibility. Of those who were eligible but did not receive antibiotics, 73.1% (*n = *1371) had minor risk factors, predominantly respiratory distress; and 28.7% (*n = *553) had major risk factors. Severe respiratory distress in a term infant, signs of shock, and meconium aspiration were seen more frequently in the group prescribed antibiotics (**Table 3**). Eligible neonates not receiving antibiotics had a lower CFR than those who did receive antibiotics: CFR 175/1000 admissions (95% CI, 156-196/1000, *P* < .001) vs 274/1000 admissions (95% CI 249-300/1000) respectively (**Table 2a**).

**Table 2a.** Comparison of CFR between Neonates Who Met National Clinical Guidelines for Antibiotics and Received Antibiotics and Those Who Met Criteria but Were Not Prescribed Antibiotics at 2 Zimbabwean Hospitals and 1 Malawian Hospital

Implementation site	Total	Sepsis predicted and antibiotics recommended (%)	% of eligible who received antibiotics	Case fatality rate among those who met criteria and received antibiotics (per 1000 admissions)	Case fatality rate among those who did not receive antibiotics despite meeting criteria (per 1000 admissions)	Fisher’s exact test
SMCH (Zimbabwe)	5878	63%	49%	273	175	*P* < .001
CPH (Zimbabwe)	1081	79%	46%	225	180	*P = *.224
KCH (Malawi)	3728	82%	43%	246	215	*P = *.048

**Table 2b.** Comparison of CFR between Neonates Who Met WHO Clinical Guidelines for Antibiotics and Received Antibiotics, and Those Who Met Criteria but Were Not Prescribed Antibiotics at 2 Zimbabwean Hospitals and 1 Malawian Hospital (Totals as per **Table 2a**)

Implementation site	Sepsis predicted and antibiotics recommended (%)	Antibiotics received (%)	Case fatality rate among those who met the criteria and received antibiotics (per 1000 admissions)	Case fatality rate among those who did not receive antibiotics despite meeting criteria (per 1000 admissions)	Fisher’s exact test
SMCH (Zimbabwe)	92%	40%	266	135	*P* < .001
CPH (Zimbabwe)	88%	46%	246	195	*P = *.089
KCH (Malawi)	77%	45%	251	227	*P = *.144

**Table 3.** Distribution of Major Risk Factors by Receipt of Antibiotics as per EDLIZ 2020 Guidelines and Meconium Aspiration Guidelines in 5878 Neonates Admitted to Sally Mugabe Central Hospital in Harare, Zimbabwe, between January 2021 and June 2022 (*n = *1366 Major Risk Factors Recorded Among 1227 Neonates)

Risk factors	Received antibiotics (*n* = 2188)	Did not receive antibiotics (*n* = 3690)	Chi-squared test (*P*-value)
Confirmed sepsis or chorioamnionitis in mother	18 (81.8%)	4 (18.2%)	.003
Seizures	24 (38%)	39 (62%)	.06
Severe respiratory distress in term infant	316 (48.5%)	336 (51.5%)	.433
Signs of shock	113 (57.4%)	84 (42.6%)	.039
Meconium aspiration	274 (66.2%)	144 (33.8%)	<.001

Considering the WHO PSBI and risk factor guidelines, 92.1% (*n = *5411) neonates met criteria for antibiotics (**Table 2b**), with symptoms of respiratory distress (*n = *3860) and lethargy (*n = *1108) seen more frequently in those eligible and not receiving antibiotics than in those who did. For WHO guidelines, age is not a criterion, meaning those with missing age values were included. The CFR in those receiving antibiotics as per criteria compared to those who did not (despite eligibility) was 266/1000 admissions (95% CI, 244-290) vs 134/1000 admissions (95% CI, 122-148, **Table 2b**).

### Zimbabwe (CPH)

Of 1094 neonates admitted during the study period, 10 had missing age values and so were excluded, giving 1084 for analysis for EDLIZ guidelines mapping. Using EDLIZ guidelines, 78.6% (*n = *852) met criteria for commencement of antibiotics, while 472 (43.5%) received them (**Table 2a**). Of 232 who did not meet the eligibility criteria, 33.2% (*n = *77) were commenced on antibiotics. Of 496 neonates with one or more major risk factors, 47.4% (*n = *235) did not receive antibiotics, compared with 62.4% (*n* = 222) out of 356 neonates with any minor risk factors. The CFR in those receiving antibiotics in line with criteria was 225/1000 (95% CI, 181-277), vs 180/1000 (95% CI, 145-229, *P = *.1) in those for whom antibiotics were not prescribed despite eligibility. Among the neonates who received antibiotics, severe respiratory distress (34.8%, *n = *164) and signs of shock (26.1%, *n = *123) were common major risk factors. Considering WHO PSBI guidelines and risk factors, 87.8% (*n = *960) neonates were eligible for antibiotics, including those with missing age values, compared with 45.6% (*n = *438) who received them. An additional 29.1% (*n = *39 out of 134) were prescribed antibiotics despite not meeting criteria (**Table 2b**). The CFR of those who did not receive antibiotics despite meeting criteria was 195/1000 (95% CI, 156-241), vs 246/1000 (95% CI, 198-302) who met criteria and did receive antibiotics (*P = *.1).

### Malawi

At KCH, 81.8% (*n = *3043) neonates met COIN criteria for commencement of antibiotics, among whom 43.4% (*n = *1322) received them (**Table 2a**). An additional 28.7% (*n = *197 out of 686) received antibiotics despite not meeting eligibility criteria. Distribution of risk factors is in **Table 4**. Most had 2 (63.7%, *n = *2377) or 3 (11.3%, *n = *421) risk factors, most commonly signs of respiratory distress, hypothermia or fever, and lethargy. The CFR in those receiving antibiotics was 247/1000 (95% CI, 220-276) vs 215/1000 (95% CI 193-239, *P = *.05) in those who met criteria but were not prescribed antibiotics.

**Table 4.** Distribution of Risk Factors Among Neonates Admitted to Kamuzu Central Hospital in Malawi as per COIN Guidelines (*n = *3729)

Risk factor	Received antibiotics (*n* = 1519)	Did not receive antibiotics (n = 2210)	Chi-squared test (*P*-value)	Total
Severe respiratory distress	1057 (70.0%)	1467 (66.4%)	0.37	2524
Hypothermia or fever (Temperature <35.5 and >=37.5)	615 (40.5%)	548 (24.8%)	<0.001	1163
Lethargy	408 (26.9%)	361 (16.3%)	<0.001	769
Shock (cold hands/feet)	258 (17.0%)	306 (13.8%)	0.02	564
Maternal risk factor of sepsis	174 (11.5%)	141 (6.4%)	<0.001	315
Convulsions	41 (2.7%)	42 (1.9%)	0.11	83
Skin abscess	27 (1.8%)	32 (1.4%)	0.44	59
Crepitations in the lungs	25 (1.6%)	10 (4.5%)	0.0002	35
Bulging fontanel	7 (0.5%)	20 (0.9%)	0.12	27
Umbilical redness	5 (0.3%)	15 (0.7%)	0.15	20
Reduced movement	8 (0.5%)	5 (0.2%)	0.13	13

For WHO PBSI guidelines, 76.8% (*n = *2865) neonates would be eligible (**Table 2b**), with respiratory distress, hypothermia or fever and lethargy more common in those receiving antibiotics, as well as similarly distributed.

### Factors Associated with Antibiotic Prescription

Prior to deletion and imputation of missing data, the risk factors and clinical signs at admission with the strongest association with antibiotic prescribing in univariable, unadjusted analyses for the cohort (*n = *10 868) included: maternal risk factors of sepsis including prolonged rupture of membranes (OR = 4.285, 95% CI = (3.613, 5.098)), offensive liquor (OR = 4.025, 95% CI = (3.125, 5.223)), being born before arrival in hospital (OR = 3.155, 95% CI = (2.61, 3.824)) and maternal fever (OR = 2.108, 95% CI = (1.214, 3.683)). Outcomes from univariable analyses for all predictor variables are included in **Table 1**.

The final multivariable logistic regression was conducted for 10 834 cases after removing missing values accounting for <1% of total cases. The following were found to be associated with antibiotics prescription based on multivariable-adjusted analysis (**Table 5**): maternal risk factors of sepsis including offensive liquor (OR = 5.27, 95% CI = (4.03, 6.89)), prolonged rupture of membranes (OR = 5.04, 95% CI = (4.23, 6.03)), born before arrival in hospital (OR = 2.65, 95% CI = (2.0-3.52)), prolonged 2nd stage (OR = 2.22, 95% CI = (1.63, 3.03)), maternal fever (OR = 2.18, 95% CI = (1.24, 3.85)), birth weight at admission (OR = 0.62, 95% CI = (0.45, 0.86)), age at admission (OR = 1.0017, 95% CI = (1.001, 1.002)). Spontaneous vaginal delivery was associated with an increased odds of antibiotics prescription compared to both elective and emergency cesarean sections and other modes of delivery. Similarly, female gender was associated with a 10% decrease in the odds of receiving antibiotics (OR = 0.9, 95% CI = (0.83, 0.88)). Odds of receiving antibiotics were higher in CPH compared to SMCH and KCH.

## DISCUSSION

In this multicountry, multicentered cohort study including data from over 10 000 neonates, we evaluated the concordance of national and international guidelines for neonatal sepsis with contemporaneous real-world decision-making by clinicians in Malawi and Zimbabwe. We demonstrated an important discrepancy between those neonates eligible for antibiotics as per documented clinical signs compared to those who actually received them. In both countries, strict adherence to guidelines would lead to the antibiotic prescription rate nearly doubling. This is a striking result considering that these neonates were admitted to a neonatal unit and so were likely to be more vulnerable than their background population. Despite differences in the national guidelines between Zimbabwe and Malawi, we found similar figures in both, with guideline-described clinical signs of sepsis frequently not taken into account.

Neither national nor international guidelines for clinical signs of sepsis were consistently adhered to in all 3 facilities over a protracted period of time. Moreover, in all settings, the CFR for those who received antibiotics was higher than for those eligible but not receiving them. Were this to be as a result of poor practice, it would be surprising to find such similar results in all hospitals. Furthermore, the CFR in those neonates eligible but not receiving antibiotics would be expected to be higher than those receiving them appropriately. It may be that clinicians are correctly identifying the more vulnerable neonates using features not included in the guideline. In our multivariable analysis, maternal risk factors, including prolonged rupture of membranes and offensive liquor, and lower neonatal birthweight were most strongly associated with a decision to prescribe antibiotics. It appears that maternal risk factors are more useful and easier to identify than clinical signs of sepsis. Postnatal age and duration of admission (pre- and postnatally) are not included in current guidelines. This is important: in the light of the changing epidemiology of neonatal sepsis in LRS to a preponderance of healthcare-associated pathogens, progressively earlier in neonatal life,^6,23,24^ the mother–infant dyad’s duration of exposure to the hospital environment may be key, and traditional definitions of early- and late-onset sepsis less relevant. Our analysis was agnostic to early- vs late-onset characterization, but these healthcare-associated factors may be features clinicians are using to triage risk.

This study is unusual in that we have detailed clinical features, management decisions, and outcomes for a large cohort of neonates in 2 countries born in low-resource facilities, with data collected prospectively at the point of admission. This has enabled examination of neonates who did and did not receive antibiotics at admission, and examination of factors associated with prescription. One previous study in Nepal found good adherence to guidelines for sepsis in 12 regional hospitals,^25^ but these were neonates who already had a preliminary diagnosis of sepsis, as opposed to recognizing guideline-described clinical signs of sepsis. Even in high-resource settings, prospective evaluations of adherence to neonatal sepsis guidelines are few as most studies to date have been survey-based.^26,27^

There are 2 key issues here: firstly, how accurately guidelines predict neonatal sepsis, and secondly their perceived utility and uptake by clinicians. Our work may call into account the former (given the lower CFR in neonates where guidelines were ignored) and clearly calls the latter. Even the most effective and accurate guidelines will not improve outcomes if they are not followed. We are currently undertaking qualitative work to explore factors impacting clinicians’ decisions to follow or ignore guidelines, which will contribute to how we build future decision support algorithms. These factors may include personal, patient-, and system-related factors and merit full exploration alongside revising guideline content.^28^

### Limitations

Although our dataset is large, it is limited to 3 hospitals, 2 of which are referral hospitals. Also, despite of receiving free health care for those under 5 years old, the cost of transportation to facilities may be prohibitive for the most vulnerable families. Therefore, our data may not be representative of practice in other facilities and all newborns within the facility catchment. Additionally, our data include only neonates admitted to a neonatal unit, who by definition are the most vulnerable. For example, this could explain the higher rate of antibiotic prescription in neonates born by spontaneous vaginal delivery within our study, demonstrating a severity bias that would not be applicable to all neonates. This important proviso should be factored in when designing clinical risk calculators. Furthermore, a small number of very sick neonates (those with a “guarded” prognosis) were excluded from analysis (99/6045), which might have contributed to skewing of results. The use of routine data in our case can pose a challenge with data quality as data are entered by busy staff with multiple demands on their time. However, inbuilt quality control is in place in all units where Neotree documentation is reviewed by seniors on ward rounds subsequent to admission and the use of mandatory data fields ensures fewer missing data. This dataset is also limited to data at the point of admission and discharge, and does not include antimicrobial duration or account for postdischarge follow-up. Work is in progress to capture neonatal outcomes postdischarge and to add daily documentation of progress, including antimicrobials. Microbiological confirmation of sepsis is not consistently available at our sites, if at all, so it is not clear that the causative pathogens would have been susceptible to prescribed antimicrobials, which might contribute to our observed results. For those newborns who died, postmortems are rarely carried out, so there is not definitive evidence of the contribution of sepsis to death: extrapolation from similar populations indicates this is likely to be substantial.^29^ The guidelines used at SMCH might include features not considered by clinicians working in other settings to represent infection (such as meconium-stained liquor), which might impact reproducibility, although this limitation does not impact our other sites. Finally, with some variables, it was challenging to match them exactly to the terms in the national/international guidelines given data are currently captured only at the point of admission using Neotree. For example, respiratory distress within EDLIZ 2020 should be assessed over time, with respiratory distress present at birth distinguished from later onset respiratory distress. Although this might have impacted on the results from Zimbabwe, the similar results from Malawi are reassuring.

Our work assessed the use of local guidelines by mapping EDLIZ and COIN guidelines for Zimbabwe and Malawi data, respectively. Given that the EDLIZ guidelines use more complex weighting for major and minor criteria, applying these to Malawi data may result in different outcome and can be explored in the future upon consultation with local clinicians. Also, there is a lack of nuance in all 3 guidelines. As the population-based cohort study within the “Simplified antibiotic regimens for neonatal sepsis” (AFRINEST) study shows, it is unlikely that all clinical features are equally as predictive of poor outcome.^12^ In high-resource settings, online calculators such as the Kaiser Permanente Score allow the user to set the background rate of sepsis incidence, and then calculate a neonate’s specific risk, with a treatment threshold set previously.^30^ Although this particular calculator is unlikely to be suitable in LRS where the incidence of sepsis is substantially higher than its source population,^31,32^ we should be aspiring to this level of nuance to identify neonates most in need of antibiotic therapy in LRS.

Alongside revision of current guidelines to factor in epidemiological changes, consideration should also be given as to how to support clinicians in adherence to revised guidelines. Challenges in guideline adherence are not unique to LRS: consistent implementation requires ongoing training, support, and senior/management-level buy-in.^32^ One strategy that has proven efficacy in high-resource settings is the use of mobile apps and decision support tools.^33^ We are aiming to emulate that strategy using Neotree to give support to clinicians caring for neonates in LRS facilities, including the incorporation of a diagnostic algorithm for neonatal sepsis.

## CONCLUSION

In this large, prospective data set, guideline-recommended clinical signs for sepsis are inconsistently used in both Zimbabwe and Malawi, with clinicians using other features for antibiotic decision-making. In both countries, adherence to current guidelines would mean a near doubling in antibiotic prescription rate, although it is not clear from our data that an increase in prescription would reduce mortality. It is time to reevaluate guidelines for identifying neonatal sepsis in LRS and to derive contextually appropriate decision support tools that will be both used and useful.

## Supplementary Material

piaf017_suppl_Supplementary_Material

## Data Availability

The dataset used for modeling is available upon reasonable request. While an open-source, anonymized research database is planned as part of the wider Neotree project, currently, sharing of deidentified individual participant data is considered on a case-by-case basis.
