# Supplementary material for: Assessing the Use of Neonatal Sepsis Guidelines and Antibiotic Prescription With Large-Scale Prospective Data From Zimbabwe and Malawi
Source: J Pediatric Infect Dis Soc. 2025 Feb 21;14(4):piaf017. doi: 10.1093/jpids/piaf017 (PMC11976057; doi:10.1093/jpids/piaf017)
Supplement: piaf017_suppl_Supplementary_Material [file piaf017_suppl_supplementary_material.docx]

**Supplementary Table 1a:** **Essential Drugs List in Zimbabwe 2020 (EDLIZ 2020) Neonatal sepsis guidelines: criteria to be used for commencing antibiotics and taking blood cultures (if available)[1]**

| Major Criteria  (Start antibiotics if any of these present) | Minor Criteria  (Start antibiotics if any two available) |
| --- | --- |
| 1. Confirmed sepsis or chorioamnionitis in mother  2. Confirmed or suspected sepsis in twin  3. Seizures  4. Severe Respiratory Distress in a term infant  5. Signs of shock | **Antenatal:**  1. Rupture of membranes >18h  2. Spontaneous Preterm Birth  3. Group B Streptococcus (GBS) sepsis in previous baby or documented GBS carriage in this pregnancy (urine or vaginal swab) |
|  | **Natal**  4. Born Before Arrival  5. Meconium stained Liquor |
|  | **Postnatal**  6. Respiratory distress that is not obviously related to:   - environmental hypothermia - “delayed transition to extra-uterine life” i.e. mild to moderate respiratory distress apparent soon after birth that is improving with time.   7. Hypoxia  8. Apnoea  9. Hypoglycaemia/Hyperglycaemia not otherwise explained  10. Temperature instability not explained by environmental factors  11. Acidosis not obviously related to HIE  12. Unexplained bleeding or thrombocytopenia  13. Mild encephalopathy/Altered responsiveness  14. Altered tone not otherwise explained  15. Feed intolerance/feeding difficulty  16. Abnormal heart rate (<90 or >160)  17. Jaundice in first 24 hours |

**Supplementary Table 1b Signs and symptoms of potential bloodstream infection as per the Care of the Infant Newborn in Malawi (COIN) guidelines[2]**

If ANY of the following are present, baby should be managed as potential bloodstream infection

| **Criteria** | |
| --- | --- |
| **Antenatal risk factors** | **Symptoms and Signs of Sepsis** |
| Fever >38 in mother | Temperature <35.5 or >37.5 |
| Rupture of membranes >18h | Bulging Fontanelle |
| Offensive Liquor | Grunting |
|  | nasal flaring |
| **Signs/symptoms of meningitis** | fast breathing |
| Drowsiness | chest indrawing |
| Lethargy/unconscious | Crepitations in the lungs |
| Persistent irritability | Umbilical redness extending to the periumbilical skin or umbilicus draining pus |
| High pitched cry | reduced movement of limbs |
| Convulsions | Many skin pustules/big boil (abscess) |
|  |  |
| **Shock (Danger signs)** |  |
| Cold hands and feet |  |
| Capillary refill time > 3 seconds |  |
| Fast and weak pulse |  |

**Supplementary Appendix: Background**

At Sally Mugabe Central Hospital, antimicrobial use is high- when last quantified, inpatient days of therapy (DOT) were between 1110-1243/1000 patient-days[3]. This is not uncommon in low-resource settings, where diagnostics are scarce and antibiotics are frequently used to ‘cover’ for sepsis in the absence of a definitive diagnosis[4]. Drivers to prescribe antibiotics are complex and multifactorial but include concerns about the prevalence of serious bacterial infections, especially in the context of a population with a high prevalence of HIV, limited training in paediatric and neonatal care leading to anxiety about missing serious diagnoses, understaffing and family expectations[4]. Although these data are specific to Zimbabwe, the themes are likely to be common in other low-resource settings.

In terms of laboratory test availability, no cultures are available at CPH. Blood and cerebrospinal fluid (CSF) cultures are rarely available at KCH, and more frequently, though still intermittently at SMCH[5, 6]. Although both blood and CSF cultures are part of national level guidelines, there is considerable stigma and fear around lumbar punctures which means they are frequently refused by families (see [here](https://www.picturinghealth.org/lumbar-puncture-africa/) videos developed to counteract that stigma https://www.picturinghealth.org/lumbar-puncture-africa/). Full blood counts are generally available at SMCH, KCH and sometimes CPH, but C-reactive protein (CRP) is rarely available.

While blood cultures are infrequently available, when they are performed, the resistance rates of bacterial pathogens is high[7]. We have previously documented blood culture positivity rates of 26% (excluding contaminants), with pathogens almost universally *Klebsiella pneumoniae*. All 54 *K. pneumoniae* isolates were resistant to ceftriaxone and 53 (98%) were resistant to gentamicin, with a case fatality rate of those newborns with culture-proven sepsis of 56%. This high rate of resistant pathogens is not unique to Zimbabwe[8-11].

While care is nominally free for those aged under 5 years old in both Zimbabwe and Malawi, if equipment, medications or tests are not currently available in the hospital the families will receive prescriptions and be told to buy the supplies for their children. This can range from cannulas to antibiotics depending on stock outs. First line antimicrobials (crystalline penicillin and gentamicin) are usually, though not always available, as are second-line antimicrobials (ceftriaxone). However, as our laboratory data has demonstrated, the most common pathogens identified at our largest site are resistant to both first and second-line antimicrobials- again, this is common in settings similar to ours[9, 11]. Carbapenems are rarely available in the hospital pharmacy, with parents being requested to purchase a course costing at least $50, likely to be out of reach for most families at our sites[12].

The out-of-pocket expenditures for vulnerable families who may have been referred into these hospitals from their homes a considerable distance away include accommodation, subsistence and transport, and may be prohibitive. Therefore although despite care being ‘free’, the most vulnerable may be unable to access it.

**Methods**

Zimbabwean guidelines are to differentiate between ‘severe respiratory distress in a term infant’ (major feature) and ‘delayed transition to extra-uterine life” i.e. mild to moderate respiratory distress apparent soon after birth that is improving with time (minor feature). Our coding reflected this, factoring in gestational age and hours after birth to distinguish between mild-moderate respiratory distress which should be counted as a minor factor (improving with time) or major factor.

References

1. Ministry of Health and Child Care Z. Essential Medicines List and Standard Treatment Guidelines for Zimbabwe., **2020**.

2. O'Hare BA-MK, K.; Mzikamanda, R.; Molyneux, L. Care of the infant and newborn in Malawi (2017) : the COIN Course - Participants Manual . University of St Andrews, **2017**.

3. Chimhini GC, S.; Madzudzo, L.; Heys, M.; Crehan, C.; Robertson, V.; Ferrand, R.A.; Sado, B.; Sharland, M.; Walker, A.S.; Klein, N.; Fitzgerald, F.C. Auditing use of antibiotics in Zimbabwean neonates. Infection Prevention in Practice **2020**; 2(2): 100046.

4. Dixon J, Manyau S, Kandiye F, Kranzer K, Chandler CIR. Antibiotics, rational drug use and the architecture of global health in Zimbabwe. Soc Sci Med **2021**; 272: 113594.

5. Neal SR, Fitzgerald F, Chimhuya S, Heys M, Cortina-Borja M, Chimhini G. Diagnosing early-onset neonatal sepsis in low-resource settings: development of a multivariable prediction model. Archives of disease in childhood **2023**; 108(8): 608-15.

6. Mwaturura T. QUALITY IMPROVEMENT OF THE PRE-LABORATORY PHASE OF BLOOD CULTURE PROCESSING IN A TERTIARY HOSPITAL IN ZIMBABWE. 41st Annual Meeting of the European Society for Paediatric Infectious Diseases (ESPID). Lisbon, Portugal & Online: ESPID, **2023**.

7. Chimhini G, Olaru ID, Fitzgerald F, et al. Evaluation of a Novel Culture System for Rapid Pathogen Identification and Detection of Cephalosporin Resistance in Neonatal Gram-negative Sepsis at a Tertiary Referral Unit in Harare, Zimbabwe. Pediatr Infect Dis J **2021**; 40(9): 785-91.

8. Iroh Tam PY, Musicha P, Kawaza K, et al. Emerging Resistance to Empiric Antimicrobial Regimens for Pediatric Bloodstream Infections in Malawi (1998-2017). Clin Infect Dis **2019**; 69(1): 61-8.

9. Iroh Tam PY, Bekker A, Bosede Bolaji O, et al. Neonatal sepsis and antimicrobial resistance in Africa. Lancet Child Adolesc Health **2023**; 7(10): 677-9.

10. Sands K, Carvalho MJ, Portal E, et al. Characterization of antimicrobial-resistant Gram-negative bacteria that cause neonatal sepsis in seven low- and middle-income countries. Nat Microbiol **2021**; 6(4): 512-23.

11. Russell NJ, Stöhr W, Plakkal N, et al. Patterns of antibiotic use, pathogens, and prediction of mortality in hospitalized neonates and young infants with sepsis: A global neonatal sepsis observational cohort study (NeoOBS). PLOS Medicine **2023**; 20(6): e1004179.

12. Chimhini G, Chimhuya S, Madzudzo L, et al. Auditing use of antibiotics in Zimbabwean neonates. Infection Prevention in Practice **2020**; 2(2): 100046.
